# P-1341. Monotherapy with Trimethroprim/Sulfamethoxazole or Minocycline for the Treatment of Stenotrophomonas maltophilia Infections

**DOI:** 10.1093/ofid/ofaf695.1529

**Published:** 2026-01-11

**Authors:** Zakery Kujat, Amima Mahmood, Alex Huang, Shannon Olson, Paige Roberts, Jing Zhao, Marco R Scipione

**Affiliations:** DMC Detroit Receiving Hospital, Detroit, Michigan; DMC Detroit Receiving Hospital, Detroit, Michigan; Detroit Medical Center, Detroit, Michigan; Sinai-Grace Hospital Detroit Medical Center, Detroit, Michigan; DMC Huron Valley Sinai Hospital, Detroit, Michigan; Detroit Medical Center-Harper Hospital, Detroit, Michigan; Detroit Receiving Hospital, Detroit, MI

## Abstract

**Background:**

Trimethoprim/sulfamethoxazole (TMP/SMX) and minocycline (MINO) are both recommended for the treatment of *Stenotrophomonas maltophilia*, however, there is a lack of comparative clinical data and IDSA guidance recommends against the use of monotherapy with any agent. The objective of this study was to assess clinical outcomes between patients treated with TMP/SMX or MINO monotherapy for infections due to *S. maltophilia*.

**Table 1.**
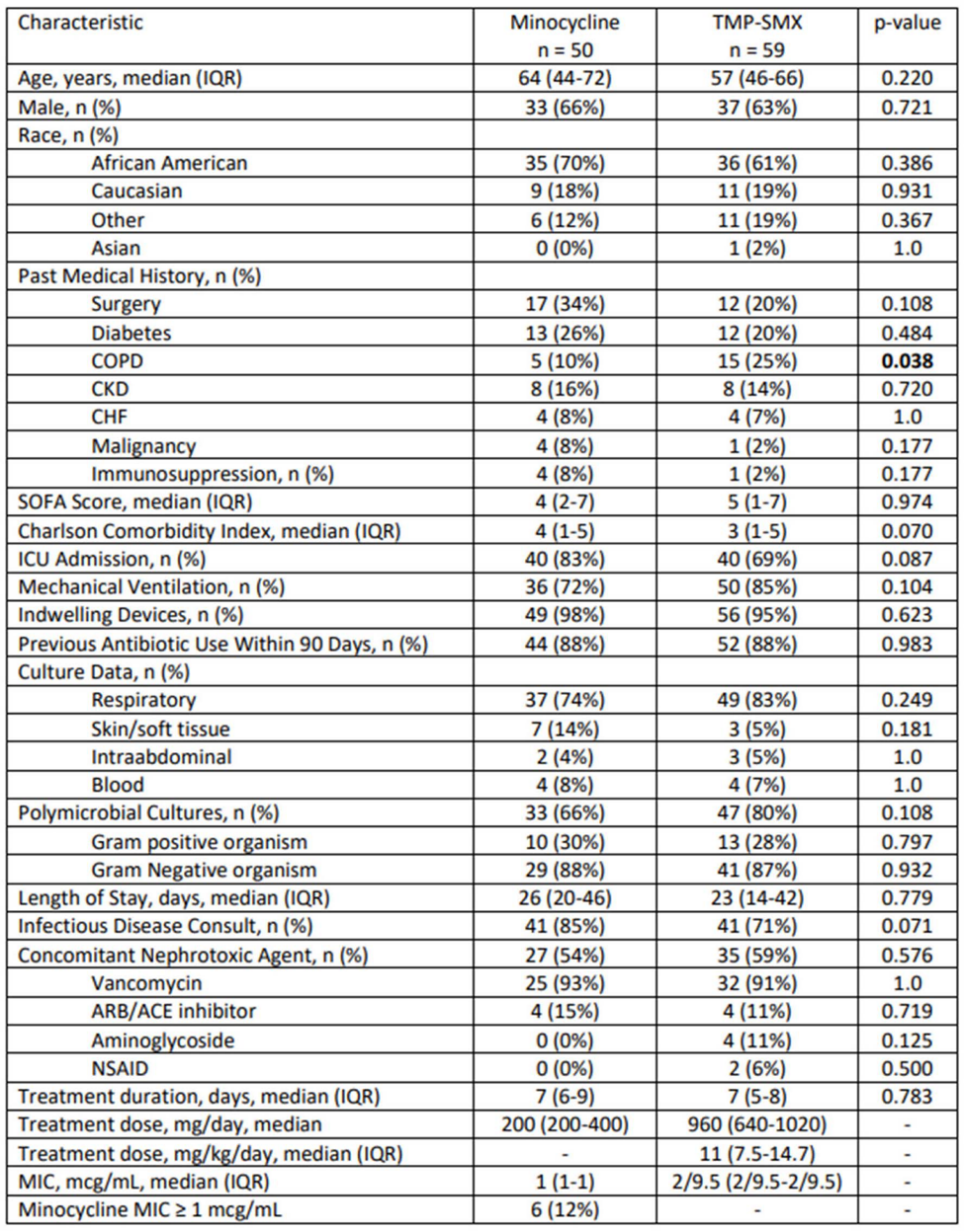
Patient and Treatment Characteristics

**FIgure 1.**
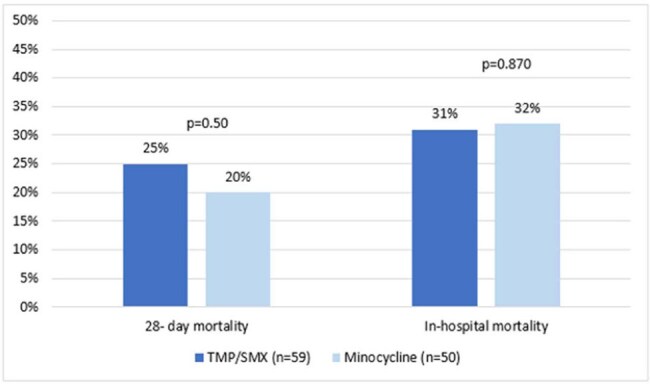
Mortality Outcomes

**Methods:**

This is a retrospective cohort study from 8/20/19 to 2/19/24. Adult patients with clinical signs of infection; a culture growing *S. maltophilia*; and who received TMP/SMX or MINO ≥ 48 hours were included. Patients were excluded if they received combination therapy; had a non-susceptible *S. maltophilia* isolate; died or were transferred to hospice within 48 hrs of treatment; or had *S. maltophilia* isolated from the urine. The primary outcome was 28-day all-cause mortality. Secondary outcomes included in-hospital mortality, microbiological cure and clinical response at the end of therapy (EOT), and acute kidney injury.

**Table 2.**
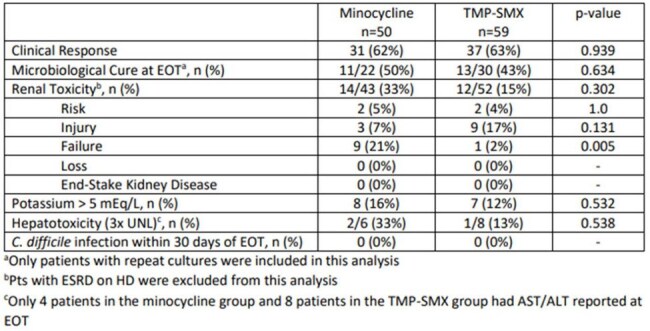
Other Clinical Outcomes

**Results:**

A total of 109 patients were included (TMP/SMX=59; MINO=50). A majority of patients were male (64%) and African American (65%). The median age was 57 years (46-66) for TMP/SMX and 64 years (44-72) for MINO, p=0.22. SOFA score (5 vs. 4, p=0.97) and Charlson Comorbidity Index (3 vs. 4, p=0.07) were similar between TMP/SMX and MINO, respectively. The most common source of infection in both groups was pneumonia (74% vs. 83%, p=0.25). A majority of cultures were polymicrobial (80% vs. 66%, p=0.11). The median TMP/SMX dose was 11 mg/kg (7.5-14.7 mg/kg), and the median MINO dose was 200mg/day. The median duration of therapy was 7 days in both groups. There was no difference in 28-day all-cause mortality (25% vs. 20%, p=0.50), in-hospital mortality (31% vs. 32%, p=0.87), microbiological cure at EOT (43% vs. 50%, p=0.63), and clinical response at EOT (63% vs. 62%, p=0.94) for TMP/SMX and MINO, respectively. Nephrotoxicity occurred in 15% of TMP/SMX and 33% of MINO patients (p=0.30) with more patients developing failure in the MINO group (2% vs. 21%, p=0.005).

**Conclusion:**

TMP/SMX had similar rates of mortality and clinical outcomes when compared to MINO for the treatment of *S. maltophilia*. Further studies should be conducted to confirm the findings of this study.

**Disclosures:**

All Authors: No reported disclosures

